# Dynamics of early stages of nose morphogenesis

**DOI:** 10.1140/epje/s10189-022-00245-8

**Published:** 2022-11-19

**Authors:** Vincent Fleury

**Affiliations:** Laboratoire Matière et Systèmes Complexes, Université de Paris Cité/CNRS UMR 7057, 10 Rue Alice Domont et Léonie Duquet, 75013 Paris, France

## Abstract

The formation of sensory organs is an important developmental and evolutionary question. In the context of regenerative medicine also, it is important to know as accurately as possible how sensory organs form. The formation of ears, eyes or nose stems presumably from tissue thickenings called *placodes* Graham and Shimeld (J Anat 222(1):32–40, 2013), Horie et al. (Nature 560:228–232, 2018) which become these organs after processes termed *inductions*. However, the origin of the placodes, the mechanism of induction and the overall face organization are not understood. Recently, it has been suggested that there is a physical principle to face organization. Indeed, it has been shown that there exists a pattern of rings and rays in the early blastula which defines the position of face landmarks, especially the ears and eyes Fleury et al. (Second order division in sectors as a prepattern for sensory organs in vertebrate development, 2021), Fleury and Abourachid (Eu Phys J E 45:31, 2022). Tensions in the sectors defined by the intersections of the said rings and rays create the actual face features. I report here that a similar situation exists for the nose. This explains the robustness of face formation in the chordates phylum. By studying nasal pit formation in the chicken embryo by time-lapse (T-L) video microscopy, I show that the nasal placode originates in a narrow sector deformed by tension forces following the biaxial pattern of rings and rays mentioned above. Cells align in the pattern and exert organized forces. Further contractions of the pattern contribute to inducing the nasal pit. The observation of the early pre-pattern of lines which locks the facial features explains readily a number of facts regarding sensory organs. Especially the existence of a lacrimal canal between the eye and the nose Lefevre and Freitag (Semin Ophthalmo l 27(5–6):175–86, 2012), or of a slit connecting the nose to the mouth, the correlation between nose, mouth and eye morphogenesis Dubourg et al. (J Rare Dis 2(8), 2007), the presence of shallow valleys on the nasal and optic vesicles, the medio-lateral asymmetry of nostrils with often a bent slit Liu et al. (PLoS ONE 12: e0181928, 2017), the uneven number of nostrils in many fish Cox (J R Soc Interf 5(23):575–593, 2008) and possibly the transition between agnatha and gnathostomes Gai and Zhu (Chinese Sci Bull 57(31), 2012): all appear under this light, geometrically straightforward.

**Graphical abstract:**

**Figure:**
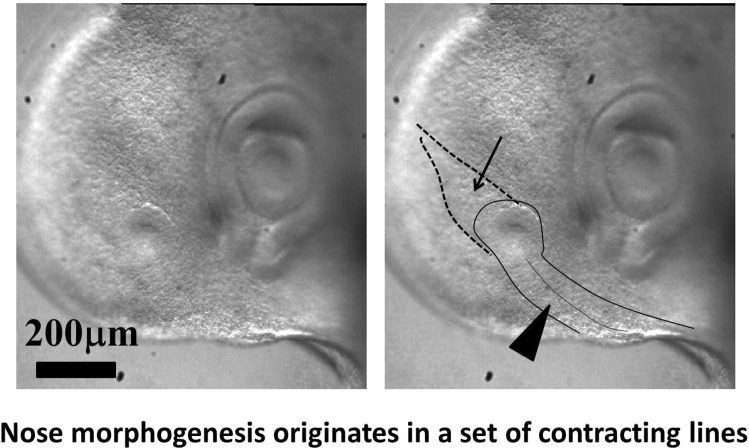
The nasal pit forms in a sector of tissue which was present on the blastodic (early embryonic stage), and which is projected onto the nasal vesicle during neurulation. The nasal pit forms along a hairpin of tissue. The top part of the hairpin forms the nares, and the bottom part a groove often visible in many animals

**Supplementary Information:**

The online version contains supplementary material available at 10.1140/epje/s10189-022-00245-8

## Main

Understanding the formation of organs is an important scientific endeavour. There exist several types of organs. Sensory organs of the head are particular in that they play a crucial role in our humanity: they are partly external, we sense the world with them, and they define our identity (an identity photography is a portrait). The biology of the face and of the sensory organs is therefore of great importance at symbolic, psychological and medical levels. In the contexts of paediatric surgery or of regenerative medicine, it is important to understand the formation of these organs with precision. The eye is certainly the organ which has attracted the highest interest, being an endless source of debate about biological complexity [10, 11]. We likely find next the ears [12, 13], and finally, the *parent pauvre* of sensory organ studies is the nose [14, 15].

**Fig. 1:**
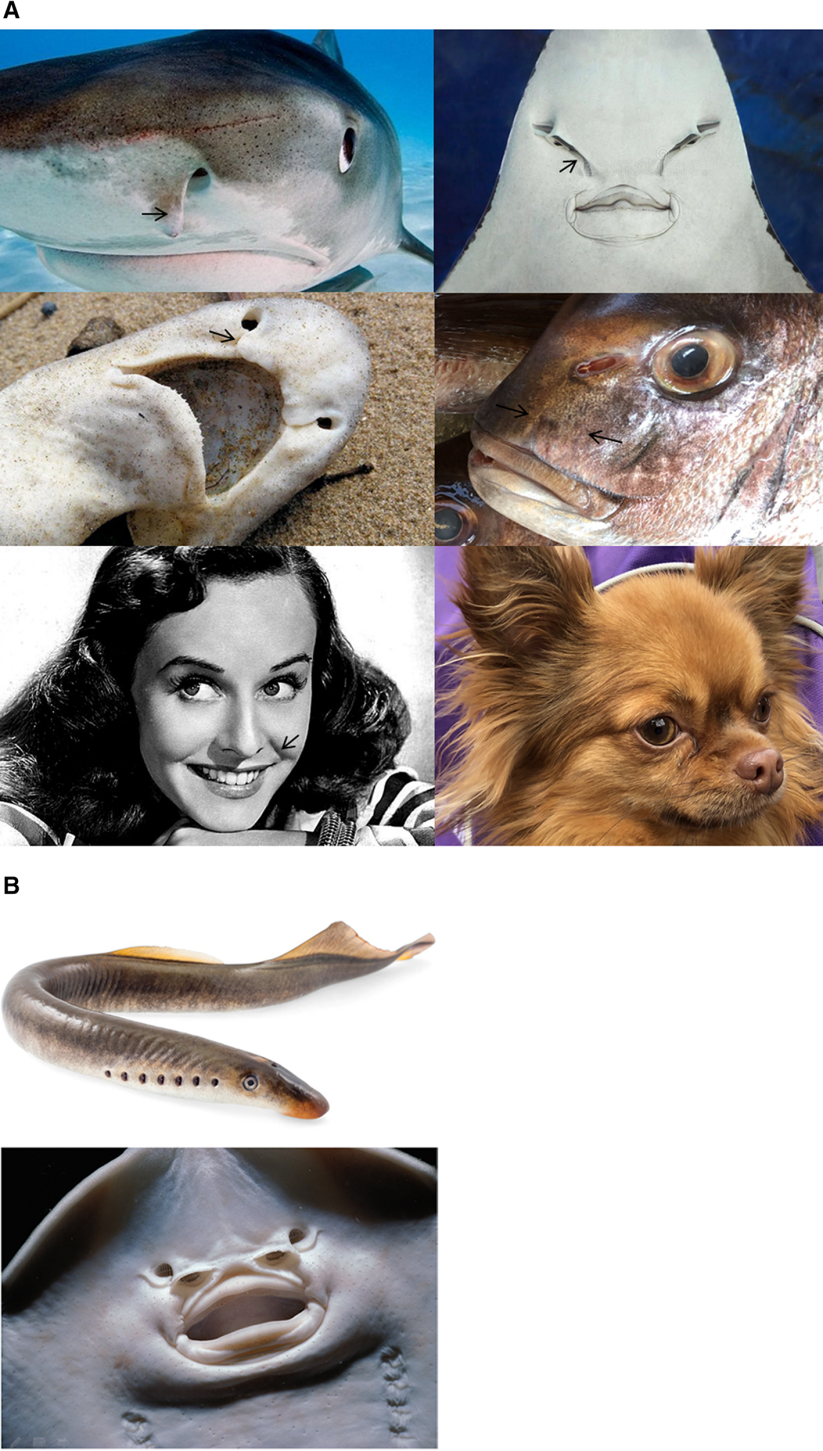
**a** Examples of nostrils. Most animals have paired nostrils. Generally, the holes present a horseshoe shape, as in sharks (top left). There also generally exists a groove (stingray Top-Right) or some visible line between the nostril and the mouth (seabream, middle right). Quite often there are slits running between the nares and the mouth (stingray, top right), or from the nare towards the eye (naso-lacrymal canal as in dogs, bottom right). Humans have a nasogenian groove (arrow in Paulette Goddard, bottom left). There exists a secondary groove below the eyes, more visible in elderlies. **b** Different nostrils shapes. Not all animals have paired nostrils. Agnatha such as the lamprey (**b** top) have a single nostril on top of the head, along the median axis. Thornback stingrays (**b** bottom) and many other fish have paired incurrent and excurrent nostrils forming a complex of four nostrils

However, it is well known classically that embryos form in two steps [16]: first, immediately after fertilization, a phase of *segmentation*, during which the zygote is regularly cleaved and called a *morula*. When the number of cells reaches a few thousands, the chicken embryo, although stratified, is flat, and it is called a *blastodisc*. Then, this blastodisc rolls up, and the embryo body acquires a 3D form as we know it, made of folded tissue. It is generally considered that sensory organs form from “placodes” [1, 2], which are paired ectodermal thickenings located around the presumptive neural territory. One finds descriptions of placodes in which such organ precursors are already positioned, at the earliest embryonic stages, in the form of round territories. However, during development, embryonic tissues undergo deformations of an extraordinary complexity [16–19] such that there is no reason a priori to expect round placodes, visible shortly before organ formation, to originate in anything like a round patch of tissue, already existing at the blastula stage. Moreover, nasal pits are rarely round, as, for example, in sharks (Fig. 1a top left) or rays (Fig. 1a top right). In many animals such as dogs they exhibit a striking bent slit pattern [7] (Fig. 1a Bottom right), which can hardly be explained by a round placode. Moreover, not all animals have paired nasal pits. The agnatha, such as the lamprey (Fig. 1b top) have a single nostril positioned on top of the head, along the median axis [20]; conversely, many fish have paired incurrent and excurrent nostrils, which makes four nostrils [8] (Fig. 1b bottom). A physicist naturally wonders whether there might be a mechanistic rationale, possibly some physical bifurcation, explaining trends in face organization, and in particular the presence of either a single nostril on top of the head, or two nostrils located on either side of the head, or four. All taxa which have bilateral nostrils present a high degree of homology, with most ancient taxa such as stingrays (Fig. 1a top right) or dogfish (Fig. 1a middle left) already having bilateral nostrils with a nasogenian groove^(^1^)^ homologous to the one seen in humans (Fig. 1a bottom left). The robustness of facial organization, and the visible pattern of lines on the animal faces such as the nasogenian groove, suggests an underlying holistic physical principle to sensory organ formation, especially the nose. Such a general principle would generate a standard prepattern, with limited variability linked to tissue visco-elasticity in the spirit of D’Arcy Thompson’s anamorphoses [21].

Alternatively, a sequence of chemical inductions in gradients, with whatever feedback loop, would be expected to generate a more arbitrary facial pattern. Stated otherwise: it seems from a physical point of view that the distribution of organs on the face is not at all arbitrary: something geometrical locks the nasal pits where they are, and nasal pits should not be expected anywhere else. It is indeed possible to position by art organs in aberrant positions (“ectopically”), by putting a bead soaked with some chemical under the ectoderm of an embryo; this assay has had a great success with limbs [22] and more recently with eyes [23]. However, this does not tell what is the “bead” in the physiological situation, and neither does it prove that nature would be able to position an organ anywhere arbitrarily by its own means. It has been shown recently that eye and ear placodes originate from bilateral trapezoidal sectors of tissue in the blastula which round off under the action of tension forces [3] to form the paired eyes and ears. I show hereafter that a similar phenomenon occurs for the nasal pits. Such a sector of tissue forming a hairpin is quite often directly visible on faces, under the nares, as for example on the seabream fish (*Pagrus pagrus* Fig. 1a, middle right) even in the absence of a nasogenian groove.

I use the chicken embryo as experimental model. The chicken head resembles fairly the mammalian head until formation of the beak begins (around day 10, HH stage 30; chicken embryo staging follows Hamilton and Hamburger stages [19]). Chicken embryology is made easier than mammal embryology by the fact that the embryos develop inside an egg. However, even in birds, observation of nasal development is difficult because the embryo develops inside a bag (the amnion, or bag of waters) with the ventral side down, and the head undergoes by the second day of development a pronounced forward flexure which contributes to hiding the presumptive nasal area and its surroundings (Supp. Fig. S1 Video 1, HH stages 15–17). This is why I have developed a sequence of dissection steps which allows one to observe the nasal area in detail, these steps are explained in the Supplementary Material (see Supp. Fig. S2). They end by a view of the embryo, from “profile” to “facial” depending on the chosen orientation. For early stages of development, I use a more classical preparation, with embryos removed from the egg, rinsed and filmed *ex ovo* on a flat Petri dish. All observations here after are in white light, with ordinary microscopes (no staining, no fluorescence, a double slit is used to enhance contrast see Supp. Material).

## Results

### In vivo observations

**Fig. 2:**
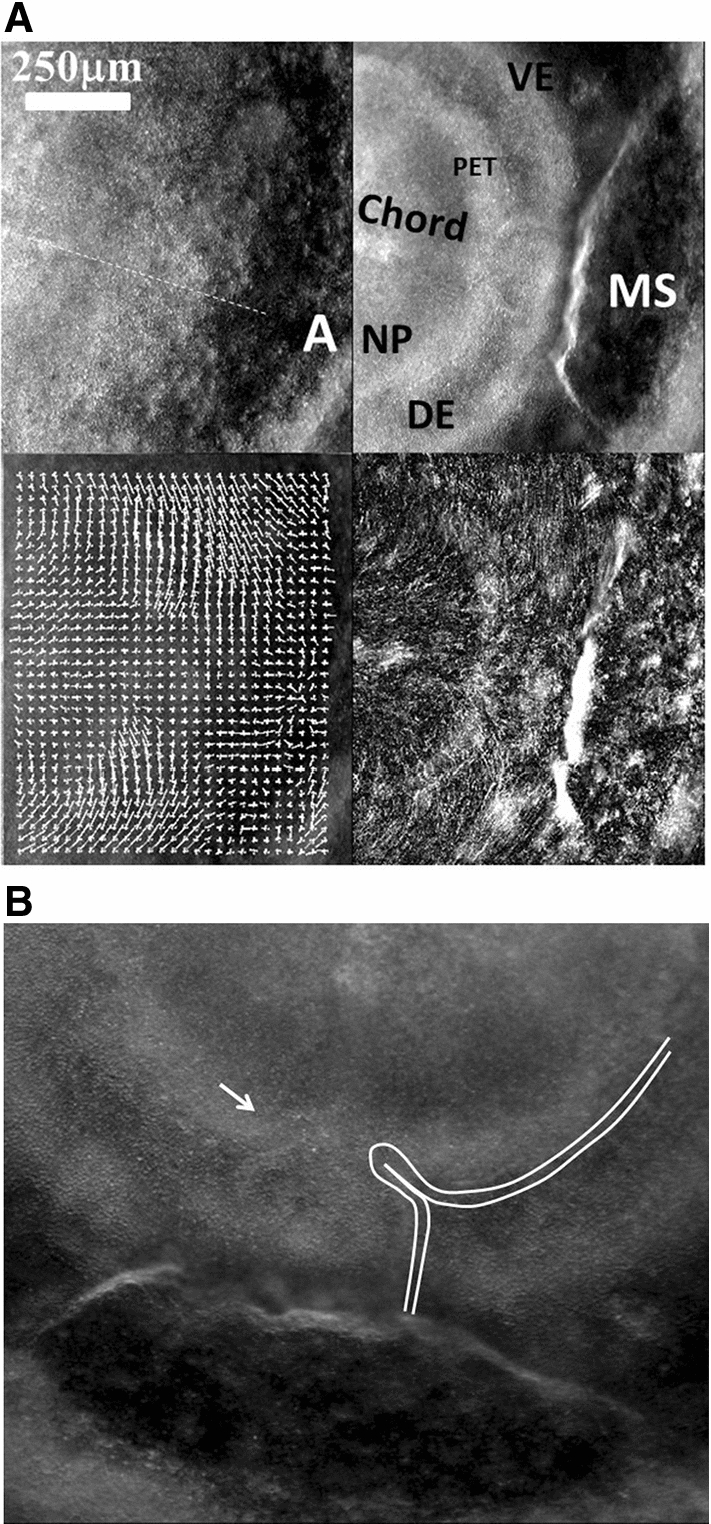
**a** Initial movements of the blastodisc (see Video 3). The chicken blastula (or blastodisc), exhibits a structure in rings and sectors. The dashed line ending at “A” shows the direction of the antero-posterior axis. The lines in ring and sectors of the blastula pattern become more visible during embryogenesis movements (top right). The lines sharpen as the embryo constricts and folds along these lines. The internal discoidal plate, visible in the top right photograph of the panel corresponds to the neural territory or neural plate (NP); one finds next the dorsal ectoderm (DE), the ventral ectoderm (VE) and the extra-embryonic organs. The foremost sector, visible here in the Top Right image by two sharp radiating lines, is associated to the mouth sector (MS). The sectors constrict, and during constriction the sectors round off to form, here, the mouth stomodeum (presumptive mouth territory). The top left photograph shows the starting situation. The bottom left shows the PIV map with the movements of the tissue, extracted from Video 3. The bottom-right image shows the actual streamlines as obtained by superimposing 30 min of images (after background inhomogeneity correction and thresholding). NP: neural plate, MS: mouth sector, PET: presumptive eye territory, DE: dorsal ectoderm. **b** Presence of a thin sector deformed in a hairpin pattern during the contraction of the mouth sector one evidences a thin sector at the ocular/oral boundary which is dragged towards the median axis and forms a hairpin

**Fig. 3:**
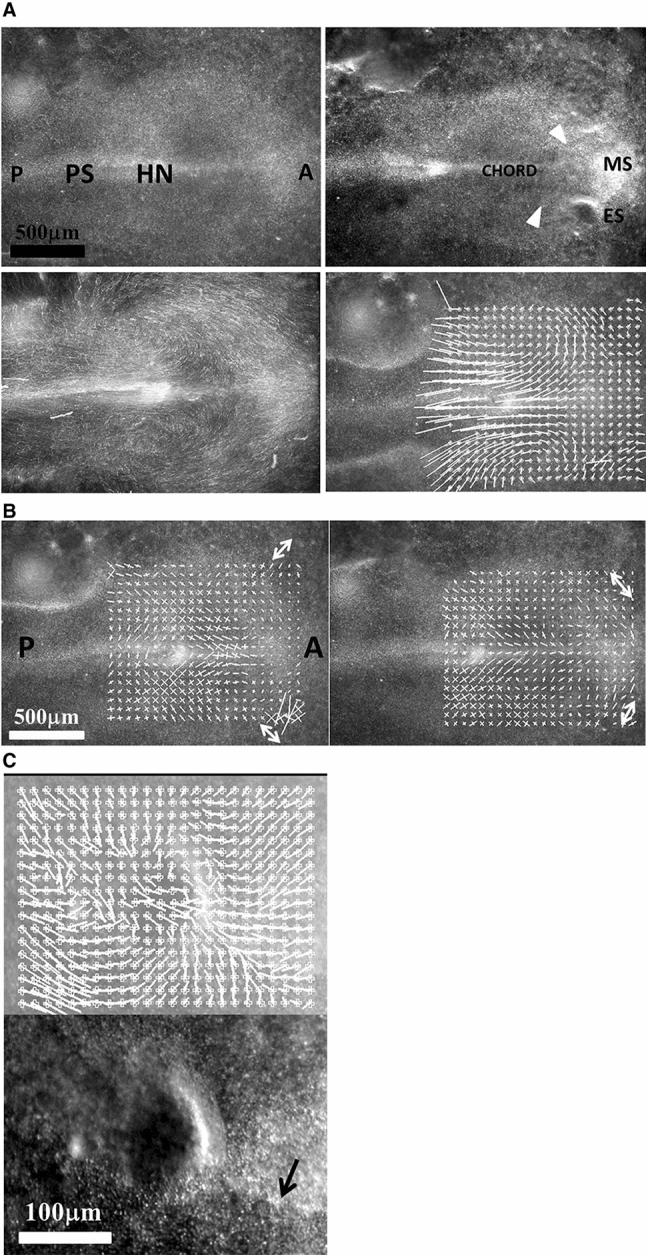
Early stages of eye placode formation. **a** The top-left photograph shows the blastodisc at start of embryo morphogenesis (approx. 20 h of development, late primitive-streak stage). A stands for anterior, P for posterior. At that stage, the avian blastodisc is flat (hence its name). **a** Top right shows the blastodisc at the onset of neural roll-up (see Video 4). Elongated sectors corresponding to nasal and ocular territories are visible (white arrowheads). Since the blastodisc is almost flat, we can extract movements and deformations by particle imaging velocimetry (PIV see Supp. Mat. PIV). We first extract the displacement field during the phase of posterior stretch by the chord. The flow map shows a vortical pattern due to the dipolar pull (Fig. 3A top right). Figure 3A Bottom Left shows the superimposition of 1 h of movement, revealing the actual streamlines. **b** Extraction of the strain field at an early stage (+ 2 h with respect to Fig. 3A top left) shows a tensile strain with principle axis oriented in the direction of the sector of the presumptive nasal area (arrow), at the corner of the mouth sector (Fig. 3B left). In this figure, the lengths of the segments are proportional to the eigenvalues of the principal strain tensor (the principal extensions). An eigenvalue of 1 corresponds to ¾ of the grid spacing. At a later stage (+ 5 h with respect to Fig. 3ATop Left) the anterior sector constricts and neurulation starts, around this moment, the principle strain tensor becomes oriented orthoradially (arrow). This shows that sources of stress have principle axis at right angles, and they are exerted in cascade: anterior contraction follows posterior traction. **c** Magnification of the eye placode at the onset of neurulation shows a domain, horseshoe shaped, which is one part of a sector of the blastula. This domain constricts along the D-V boundary, with a rotatory movement oriented towards the inside of the placode. The black arrow points to the Dorso-Ventral boundary

**Fig. 4:**
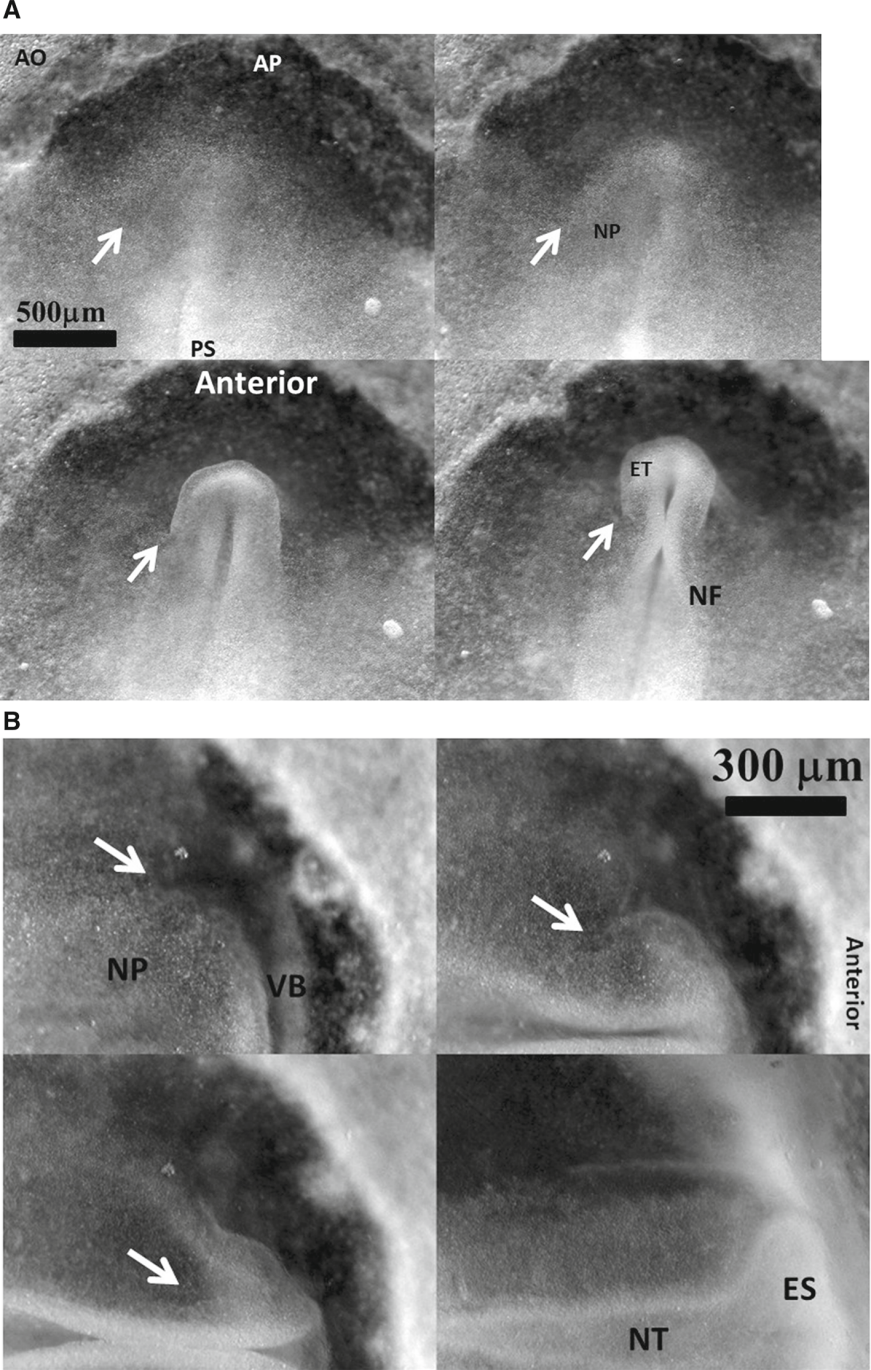
Early stage of eye formation. **a** (from Video 5) shows the deformation during early stages of neurulation. The neural territory buckles and forms a first bump locked at the boundary of the sector corresponding to the presumptive eye territory (arrows). **b** (from Video 6) The eye stalk starts to expand sideways exactly in front of the bump in the neural folds, locked by the boundary of the sector (arrows). PS: primitive streak; ET: Eye territory; AP area pellucida; AO: area opaca; NT: neural tube; NP: neural plate; NF: neural folds; ES: eye stalk; VB: ventral boundary

**Fig. 5:**
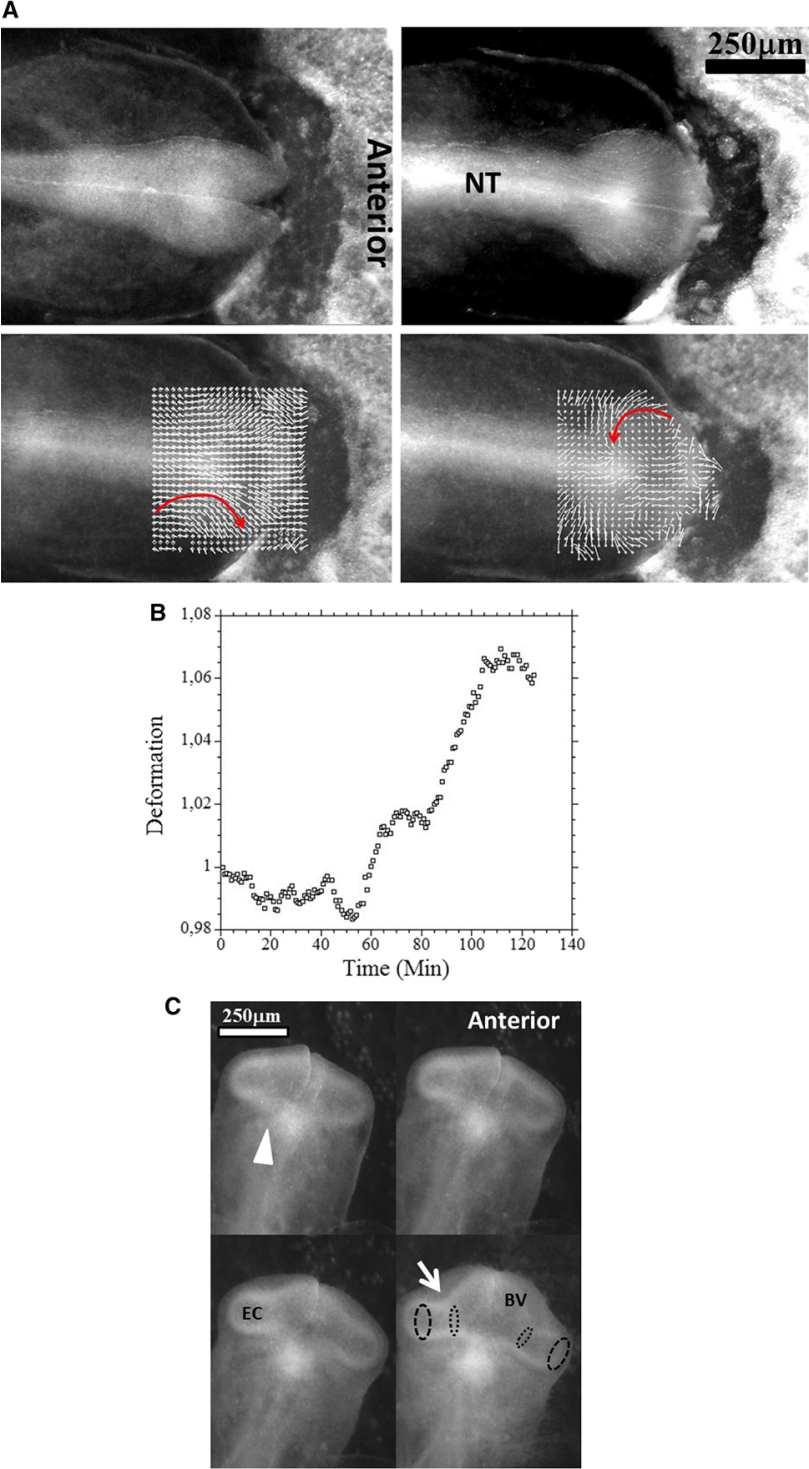

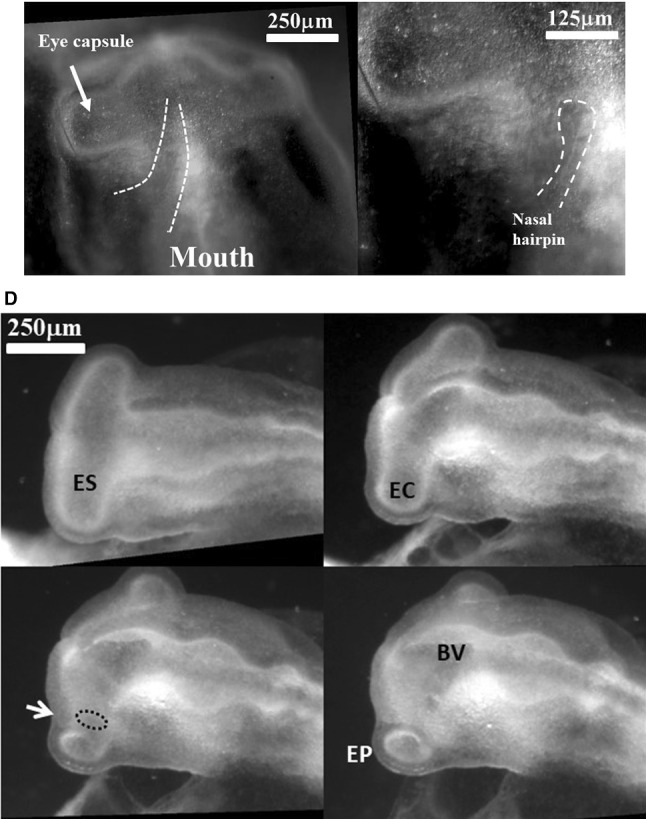
Physical winding of the eye territory during neurulation **a** (from Video 7). By the end of neurulation the neural tube (NT) closes. The extension of the tube causes a forward movement and rotatory recirculation of the eye tissue which undergoes a bilateral dipolar tissue flow (red arrow). The dipolar flow orients the eye stalk expansion in a lateral direction. The movement becomes more posterior as the tissue winds. Top left the neural tube just before closure. Top right: superimposition of frames during 2 h of movement show the actual streamlines winding laterally. Bottom left extraction of speeds by PIV just after closure of the tube shows the lateral movement. Bottom Right, two hours later, the movement is more rotatory (arrow) as a consequence of physical winding of the tissue. **b** The closure of the neural tube correlates with a sudden dilation of the brain vesicle and of the oculo-nasal territory, and even of the entire head, and to an acceleration of the eye extension (see Videos 9, 10 and 11 in which the acceleration of brain dilation is quite visible). The graph shows the deformation rate between the presumptive eye and nose territory, before and after notopore closure extracted from Video 9 (4 frames of which in** c**). **c**. Figure 5C top (from Video 9): Early stage of eye stalk extension, as seen from underneath at Mag. 4X, shows the constriction of the basis of the stalk as it expands (arrow); also, the nasal sector is visible (arrowhead). After posterior winding of the eye capsule, the eye placode will correspond to the ectoderm facing the eye capsule (large dots line). BV: brain vesicle, EC: eye capsule. Figure 5C bottom (from Video 10): At Mag. 5X and 10X, the formation of the nasal hairpin, in between the eye and mouth territories, is visible. The nasal hairpin forms a loop, the centre of which will define the nasal placode and pit. **d** (from Video 12). A ¾ dorsal view of eye stalk expansion shows the flattening of the eye stalk against the dorsal ectoderm, and the progressive constriction of the eye stalk (arrow). The flattening against the ectoderm forms the round ocular placode. ES: eye stalk, EP: eye placode, BV: brain vesicle

**Fig. 6:**
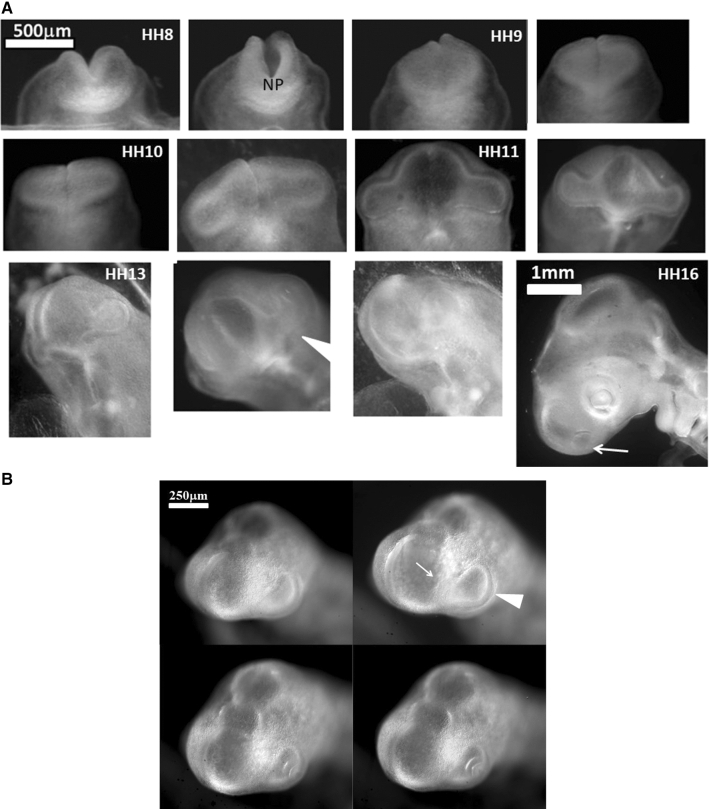
Formation of the eye. **a** Table of images of head stages since neurulation and until nose pit formation. One observes a winding of the eye stalk oriented in the posterior direction such that the root or basis of the eye stalk (the orifice opposite to the eye territory, located at the other end of the eye stalk tunnel and opening into the brain vesicle) becomes offset from the eye capsule (arrowhead). The so-called eye placode is the result of the flattening of the eye capsule against the surface ectoderm. In the last image the arrow points to the nasal pit. A sector of tissue with different contrast is also visible between the nasal pit and the median axis (NP: Notopore). **b** A ¾ facial view shows the ocular placode (arrowhead). The placode has an oblate form with an asymmetry. The eye territory points towards the oral and nasal territories. (Montage obtained from the time-lapse of eye formation in Video 14)

During early stages of embryo morphogenesis (HH5), one classically observes a strong posterior pull of the median axis which forms the chord of the chordates and triggers the roll up of neural tissue to form a tube (see Video 2). During this event, all anterior parts of the embryo are positioned [17, 24]. If we look in detail the anterior area prior to neural roll-up, we observe the deformation by contraction of one anterior sector of tissue which defines the position of the mouth (Fig. 2a,b, Video 3). During chord extension, the contraction of the mouth sector continues, but other sectors become visible (Fig. 3a, Video 4). One portion of sector is the presumptive eye territory, it has a horseshoe shape at this stage (Fig. 3b), bounded by the edge of the ventral ectoderm (see visible line boundary in Fig. 3b, arrow), which also constricts. During early neurulation, both the eye and the mouth sectors are stretched posteriorly by chord extension: they form visible elongated trapezoidal sector patterns (arrowheads in Fig. 3a, see also annotations in Video 4). At the beginning of neurulation (HH6–8), the eye territory rolls up, and it forms a bump locked by kinks or valleys in the neural tube, corresponding to the boundaries of the sector (Fig. 4a, b, Videos 5, 6). The eye balloons out laterally from the neural tube in front of this sector (HH9–12, Fig. 4a, b, Videos 7, 8, 9,10). The onset of lateral extension (HH9, Video 7) coincides with convergent-extension movements [25, 26], which impart a dipolar rotatory movement (i.e. laterally, sideways and away from the body axis) to the eye territory [27] (Fig. 5a). Most importantly, the closure of the neural tube (HH10) coincides with a sudden dilation of the head features (Fig. 5b, see Video 9 and Video 11; in Video 11 the arrowhead points to the closing neural tube, the apex of the closure is called notopore). As the brain vesicles balloon out, the eye stalk extends (HH11–12, Videos 8, 10, 12 Fig. 5c, d, Fig. 6). Flattened by brain dilation, the eye territory swerves its way towards the posterior direction where it forms an oblate capsule (the presumptive eye proper) (Fig. 6a,b, Videos 12, 13), in Fig. 6A the arrows point to the posterior winding of the presumptive eye capsule. The buckling of the eye territory (HH15, Videos 1, 13) generates a propagating fold which is oriented towards the presumptive mouth corner. Stated otherwise, the eye invagination movement is biased and it has an almond shape oriented towards the mouth corner (this will be important later), corresponding in the initial situation to the kink pointed by the arrow in Fig. 2b.

The eye forms by contraction, delamination and a double invagination of the surface layer which gives the lens, and of the ectoderm of the eye capsule which forms the eye ball (see Video 8 in Ref. 3). These events have been documented before [3, 4, 28].

**Fig. 7:**
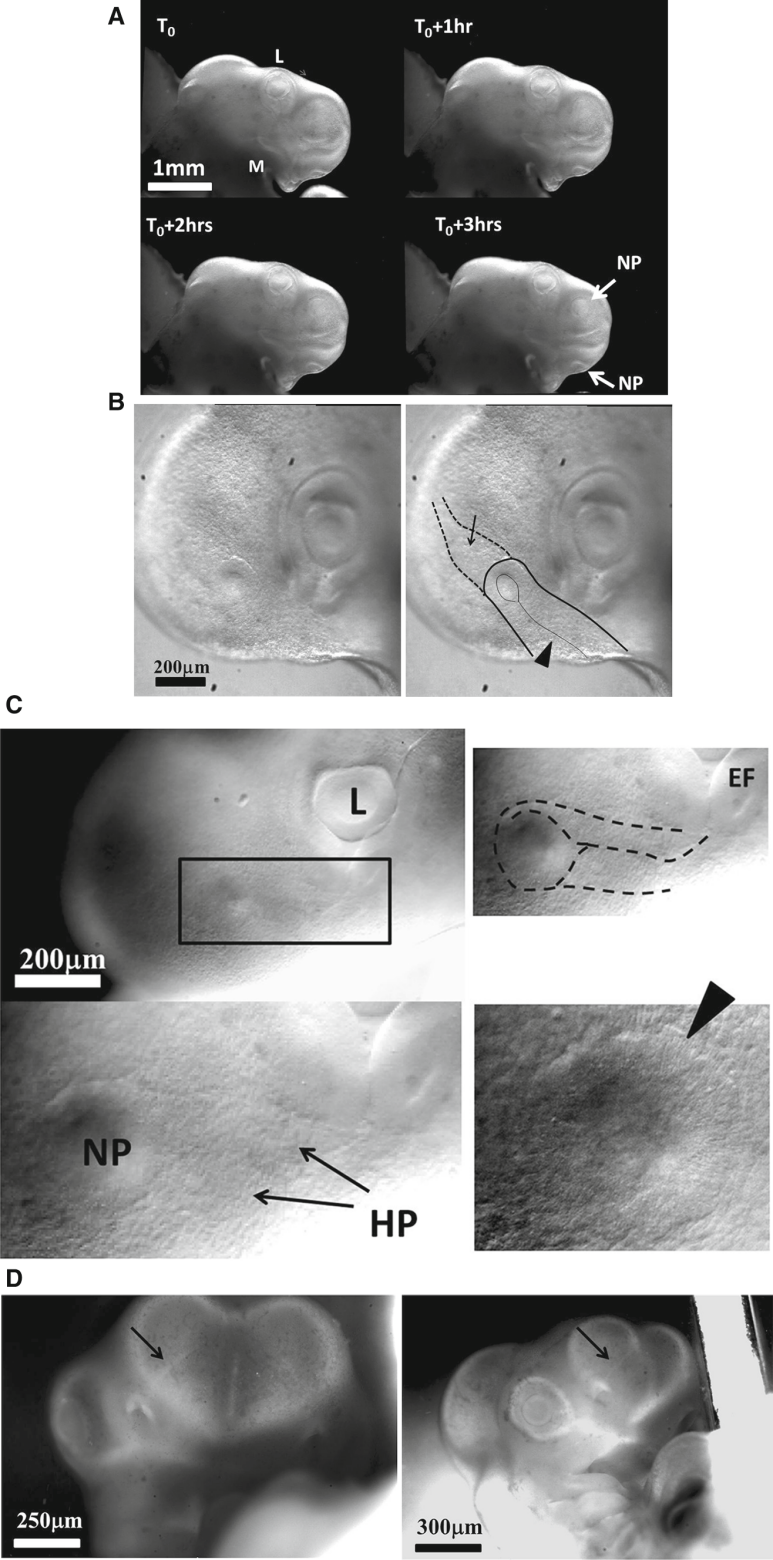
Formation of the nasal pit. **a** Formation of the nasal pit as observed at magnification 2X (from Video 15) Formation of the nasal pit is a quite rapid process. After a sudden delamination (see Videos 15–19), the nasal pit becomes well visible in approximately one hour. It is accompanied by complex movements in the oculo-nasal interspace (NP = nasal pit; M: mouth; L: lens). **b** From Video 17: During nasal pit formation one observes a sector of tissue visible above the nasal pit (arrow), and a territory in the form of a hairpin around and below the nasal pit (arrowhead). **c** Hairpin of tissue visible below the nasal pit which constricts strongly (see Videos 18, 19 and 20). Along the forming nares, the cells register radially (they stack parallel, black arrowhead); the depth of the nasal pit starts to be visible by topographic contrast in front of the registered cells. The nasal area and the ocular area converge towards the mouth. The oculo-nasal corner where a lacrimal canal exists in the adult reflects the situation at start (see Fig. 2B and also Videos 2 and 9). (NP = nasal pit; EF = eye fold; HP = hair pin). **d** 24 h later, the nare is well formed, it has a horseshoe shape and it is connected to the median axis by a thin thread (arrow). The thin thread is associated with a shallow valley on the nasal vesicle (**d** right)

In the next 6 h after eye formation, the nasal pit forms (Fig. 7a). Video 15 shows the formation of the nasal pit as seen under a binocular at Mag. 2X. We observe a nonlinearity of nasal pit formation: a rapid snap contraction followed by a relaxation during nare invagination. Video 16 shows the moment of first appearance of a nare ridge at Mag. 3X; it shows a correlation between a contraction of the nasal territory, and onset of nare formation. Videos 17 shows the formation of the nasal pit ridge at Mag. 4X (Leica binocular), Videos 17, 18 and especially 19 show at mag. 10X (Nikon Eclipse microscope) that the contraction of the nasal area precedes nare invagination. Video 20 shows a magnification of nare ridge invagination at the best possible resolution with our system. It shows that nare opening follows the nasal contraction and that cells are regularly stacked radially along the nare ridge prior to opening of the nare.

In the videos, especially Videos 17 and 18, we can see an organized tissue structure forming a physical continuity: a sector of tissue is well visible *above* the nasal “placode” in video 17 (dashed lines, arrow in Fig. 7c), and a hairpin of tissue is visible *under* the nasal placode (line and arrowhead in Fig. 7b, dashed line and arrow in Fig. 7c). A furrow is often visible (thin line pointed by arrowhead in Fig. 7b right, thinner dashed line in Fig. 7c right, see Video 18). The nasal territory in Fig. 7c, at the moment of nare opening, is similar to the one already present just after neurulation (Fig. 5c, end of Video 10).

During formation of the nasal pit, it is seen that cells register (“align” or “stack”) along the edge of the presumptive nostril edge or ridge (arrowhead in Fig. 7c from Video 17, and also in Video 19 –not the same embryo). This cell stacking is also visible in the eye and the ear (see Video 20 of ear formation, or Fig. 4 in Ref. 4). The nasal pit edge follows a horseshoe shape oriented in the direction of the mouth-eye corner. This horseshoe is actually the top part of the hairpin of tissue visible in the tissue, in the same spirit as the ear is the top part of an otic hairpin of tissue [4].

At the end of all this process, the horseshoe shape of the nostril is quite recognizable (HH20, Fig. 7d), and there is a thin thread running from the nose all the way up to the top of the nasal vesicle. This thread connecting the nostril to the median line makes a shallow furrow separating the ocular and nasal vesicles (Fig. 7d Right).

In summary, the nasal territory is not at all round, it rather has a hair-pin shape from start, a hairpin laying inside a sector. This hairpin shape is deformed by the ballooning of the nasal vesicle, but it is still present at all developmental stages. Returning to Videos 3, 4 and 10 right, we see that the position of this hairpin corresponds to a thin sector located between the presumptive eye sector and the presumptive mouth sector (arrow in Fig. 2b). During mouth contraction, the sector bounded by the presumptive mouth and presumptive eye corner is flexed and folded towards the median axis, thus generating a hairpin of tissue located inside a sector (the white hairpin overlaid in Fig. 2b). This sector is also visible in Videos 7, 9, 11 and 22, where it appears as a darker wedge, and especially in Video 10, where the hairpin is quite visible by the end.

## Dynamic analysis

**Fig. 8:**
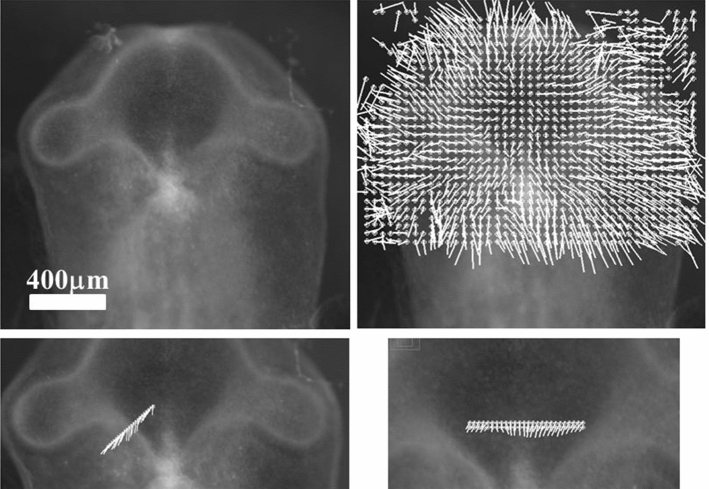
Evidence of a linear force along the lines in the embryo (from Video 13). After closure of the notopore, the brain vesicles balloon out. The PIV map shows a radial expansion of the vesicles. However, if one measures in more detail the speeds perpendicularly to the lines visible on the embryo, one sees that there is a maximum of speed along the lines, with a gradient of displacement away from the lines. This implies a shear force exerted along the lines visible on the embryo head. As the brain vesicle is quasi spherical, gradients in azimuthal movements are actual physical gradients (not projection artefacts)

In the videos above of nasal pit formation, we observe movements. These can be extracted by Particle Imaging Velocimetry (PIV, see Supplementary Material PIV). First, we observe that the brain vesicles dilate (Fig. 8a, from Video 12). Furrows or valleys appear as these vesicles dilate: something linear hinders the dilation. These valleys on the brain vesicles already exist as visible kinks during neurulation (Videos 2, 4), and they lock the position of the eyes and of the nose. In a late stage embryo, it is common knowledge that these furrows also serve as mechanical cue to position the main blood vessels, as seen classically in a mouse embryo E.13.5 (Supp. Figure 3). When following in more detail the expansion of the brain vesicles we observe a gradient of movement (i.e. shear) parallel to the furrows with a maximum inside the furrow (Fig. 8b).

**Fig. 9:**
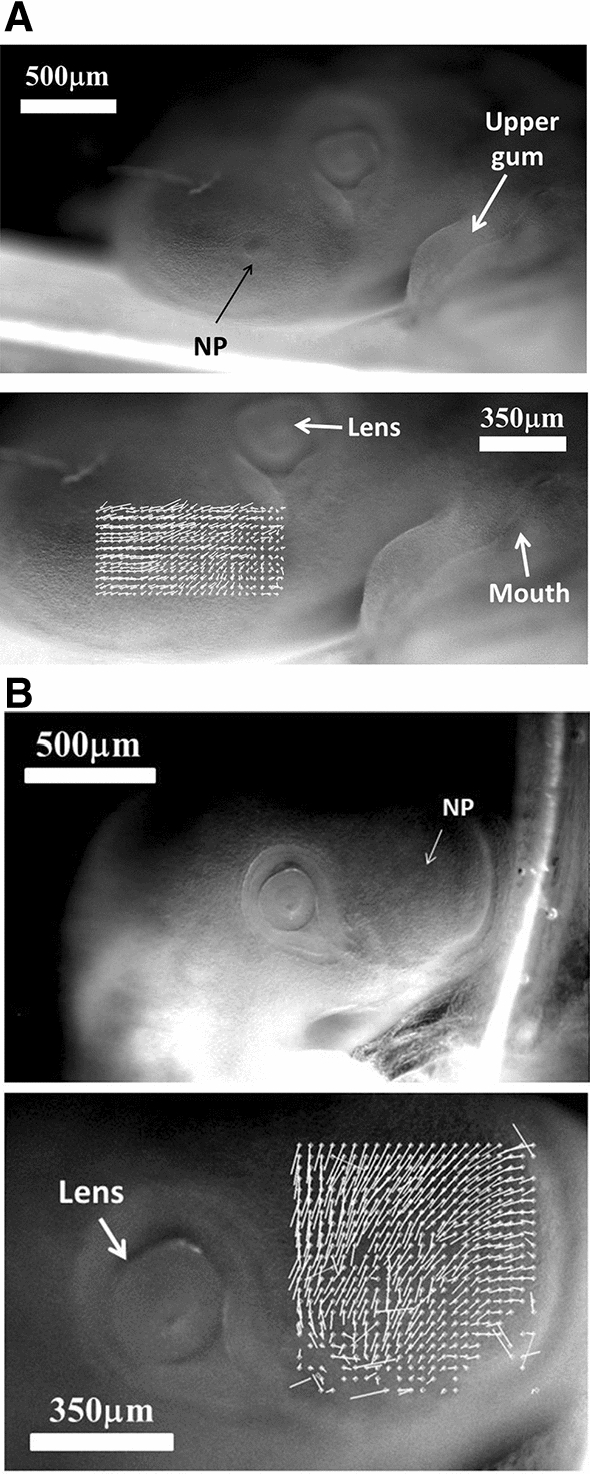
Spatial analysis of the nasal territory movement. During nares morphogenesis, there exists a movement of the nasal territory towards the eye. The plates 9A and 9B show the movement of the oculo-nasal area (rescaled to visible sizes) extracted by PIV, in the moving frame of the eye. It is seen that the entire presumptive nose territory is dragged towards the eye by a contraction of the oculo-nasal interval. (NP = nasal placode). Similar movements are very clearly visible in Videos 16 to 20. The close relationship between the nose and the eye originates in the hairpin fold visible in Figs. 2B and 5C

**Fig. 10:**
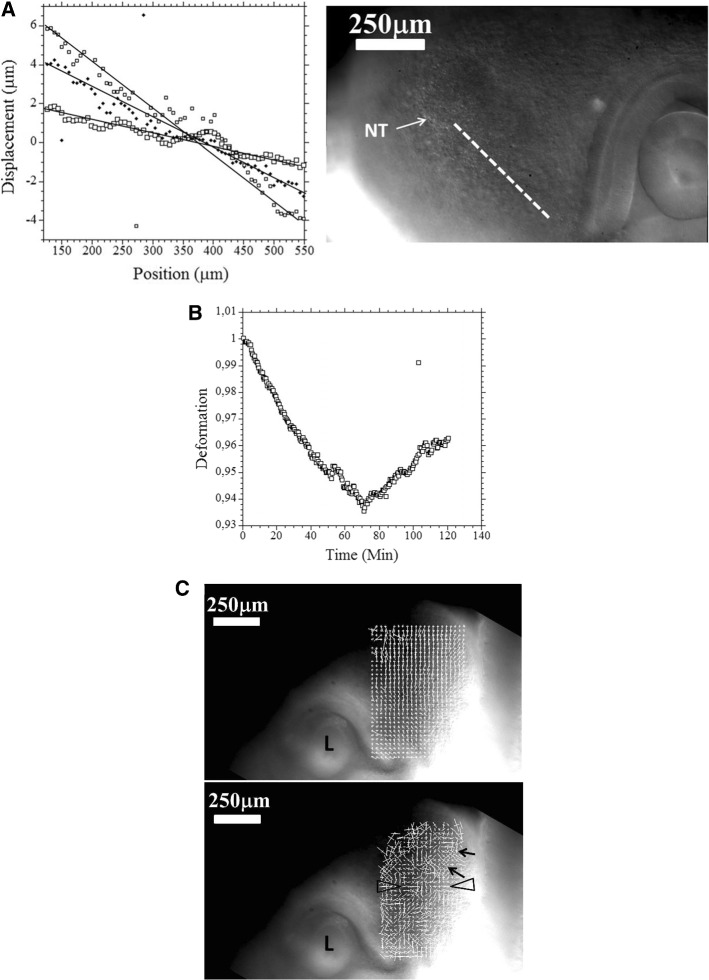
Quantitative analysis of the oculo-nasal contraction. **a** A fine spatio-temporal analysis of the movement in the nasal territory shows a spatial decay (descending slope) of the displacement from the nose towards the eye, revealing that the strain is contractile (a dilational strain corresponds to an ascending slope instead). The temporal variation (3 different times here T0, T0 + 20Min, T0 + 40Min.) shows that the strain increases with time (the slope of the gradient of displacement increases), as visually obvious on the Videos 15 to 19. To the right the area of interest, the dashed line shows the segment along which the analysis is done (NT = Nasal Territory). **b** The temporal analysis of the contraction (by following by PIV the distance between a point close to the presumptive nose, and a point further down the nose sector: the distance shrinks) shows that the contraction increases (the distance between points gets smaller) until there is a sharp relaxation. **c** Analysis of the strain at the beginning of the contraction of the oculo-nasal territory. The top image shows the displacement map, and the bottom image the strain map. The displacement is oriented towards the eye corner. The strain shows a circumferential orientation of the principal strain tensor (arrows) around the presumptive nare (axis aligned radially and orthoradially). Below the presumptive nare, the principal strain tensor is oriented with the long axis perpendicular to the oculo-nasal movement, which means that the tissue is compressed towards the eye corner (not the same embryo as **a** and **b**)

During formation of the nasal pit and nares, we repeatedly observe a strong contraction, located between the presumptive nare and the eye (*N* = 10 embryos, 100%). (Fig. 9a, b, c, Videos 15 to 20). When the movement is observed in detail, it is seen that there is a movement of the presumptive nasal territory towards the eye-mouth corner. The movement we observe is a contraction of the hairpin (Fig. 10a). The deformation in the hairpin increases during time until there is a relaxation (Fig. 10b). This contraction and relaxation correlates with the sudden appearance of the nostril edge (Videos 15 to 19), the nostril edge forms by contraction of the ridge, which causes invagination and opening of the nares (see carefully Video 20).

**Fig. 11:**
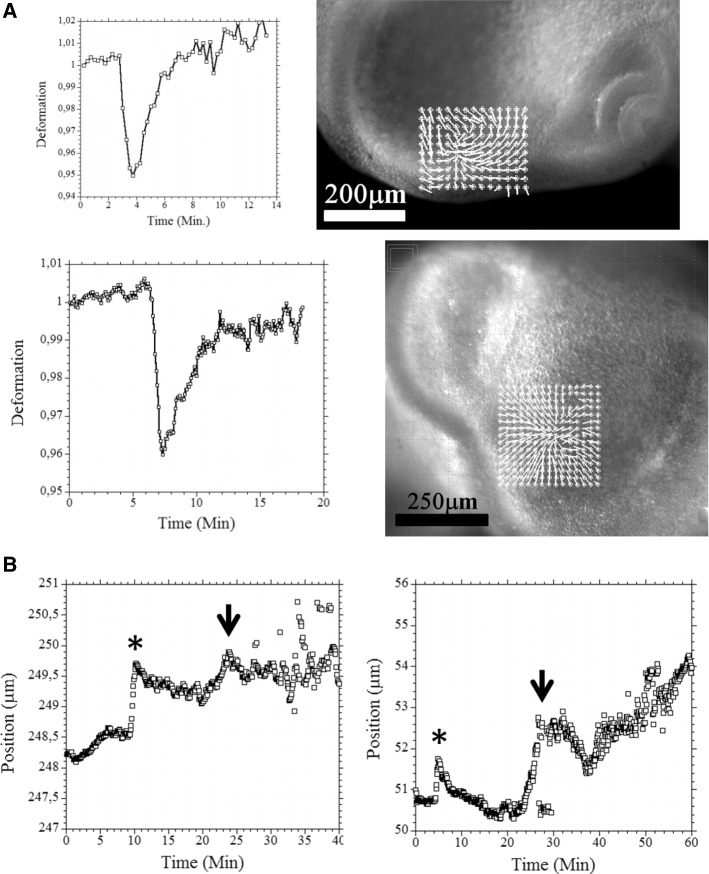
Excitability of the oculo-nasal tissue. **a** The oculo-nasal territory shows sporadic twitches of contraction of amplitude 5% and period 10 Minutes (from Video 23 and another similar Video in a different embryo). The graphs show the temporal behaviour of typical contraction twitches, as measured by PIV (by following the distance between reference points), and the images to the right show the foci of movement. The contraction twitches reveal a pattern of relaxation (rapid almost linear contraction followed by a relaxation) intrinsic to the dynamics of the tissue. **b** The contraction twitches are in fact 3D and they flex the ectoderm towards the underneath layers prior to nasal pit formation (see Videos 24, 25). The graph **b** left shows the movement of the neural ectoderm during such a twitch. The first peak (star) shows the direct mechanical effect of the twitch (flexure of the surface, the data shows the depth of the movement, the higher the peak the deeper the flexure), but a detailed PIV monitoring shows that the twitch excites an endogenous response of the neural ectoderm (arrow). (Videos 24, 25), while **b** right shows a similar twitch in the ocular area, prior to lens formation (Video 25, 26)

Sporadic endogenous contraction twitches are observed in the oculo-nasal area (see Video 23 Fig. 11a) prior to nose morphogenesis. As the period of contraction is a couple of minutes, the rate of frames acquisition must be in the 5 s range in order to resolve these contraction movements in detail. These twitches are the manifestation of an excitable behaviour, which we have found elsewhere during embryo development [29], especially in the ear sector, which also exhibits sporadic contraction twitches preceding morphogenesis [3]. These sporadic contractions are not just in plane, they flex the ectoderm towards the interior (11B, Left, Video 24), this is also observed at the moment of eye morphogenesis (Fig. 11b right, Videos 25, 26 shows the flexure triggered by the twitch). When we follow quantitatively by PIV the movement of flexure, both in the nasal area: Fig. 11b Right, and in the eye area: Fig. 11b left, we see that the superficial twitch compresses the underneath tissue (star) and it is followed by a delayed response of the underlying tissue which responds to the ectodermal twitch by its own movement of flexure (arrow).

## Discussion

These observations suggest a physical, mechanistic, rationale to nasal pit origin. First of all, the blastodisc is structured in sectors and rings, as reported earlier [3, 4]. During neurulation, the chord extends: the boundaries of the sectors forming physical landmarks are stretched posteriorly. Careful observation of the Videos such as Video 2 shows that such landmark lines are present on the blastula surface that they are stretched and that they hinder tissue expansion. This forms the kinks or furrows in the neural tube surface. The ocular territory is locked by such kinks at the boundaries of one sector (Fig. 3). This localizes the outward bulge forming the eye territory during brain dilation. The extension of the eye capsule takes a rotatory start under the convergent-extension movement [25–27] (Fig. 4). The presumptive eye territory swerves between the surface ectoderm and the dilating neural tube (Video 13, Fig. 11a).

Not surprisingly, the anterior sector is quite symmetrical, and it is associated to mouth formation and head flexure (Video 27) as reported before [4]. However, if we look in detail the situation in between the eye sector and the mouth sector, we see that the tissue at the blastula stage (hence a rather 2D layer) undergoes an in-plane flexure or folding movement under both the contraction of the mouth sector and the pull of the chord, which creates a deep kink which is the presumptive hairpin of the future nasal pit, with its own sector connecting it to the median line. Actually, this situation is directly visible in many fish such as the dogfish, as if the dogfish development were arrested earlier than the chicken development (Fig. 1a middle left). While in the dogfish, there is an actual furrow between the nares and lips, in the seabream fish the furrow is healed, but a hairpin of tissue connecting the lips to the nares is still very well visible in the adult (Fig. 1a middle right, please observe carefully the tissue in between the nare and the upper lip of this fish.)

**Fig. 12:**
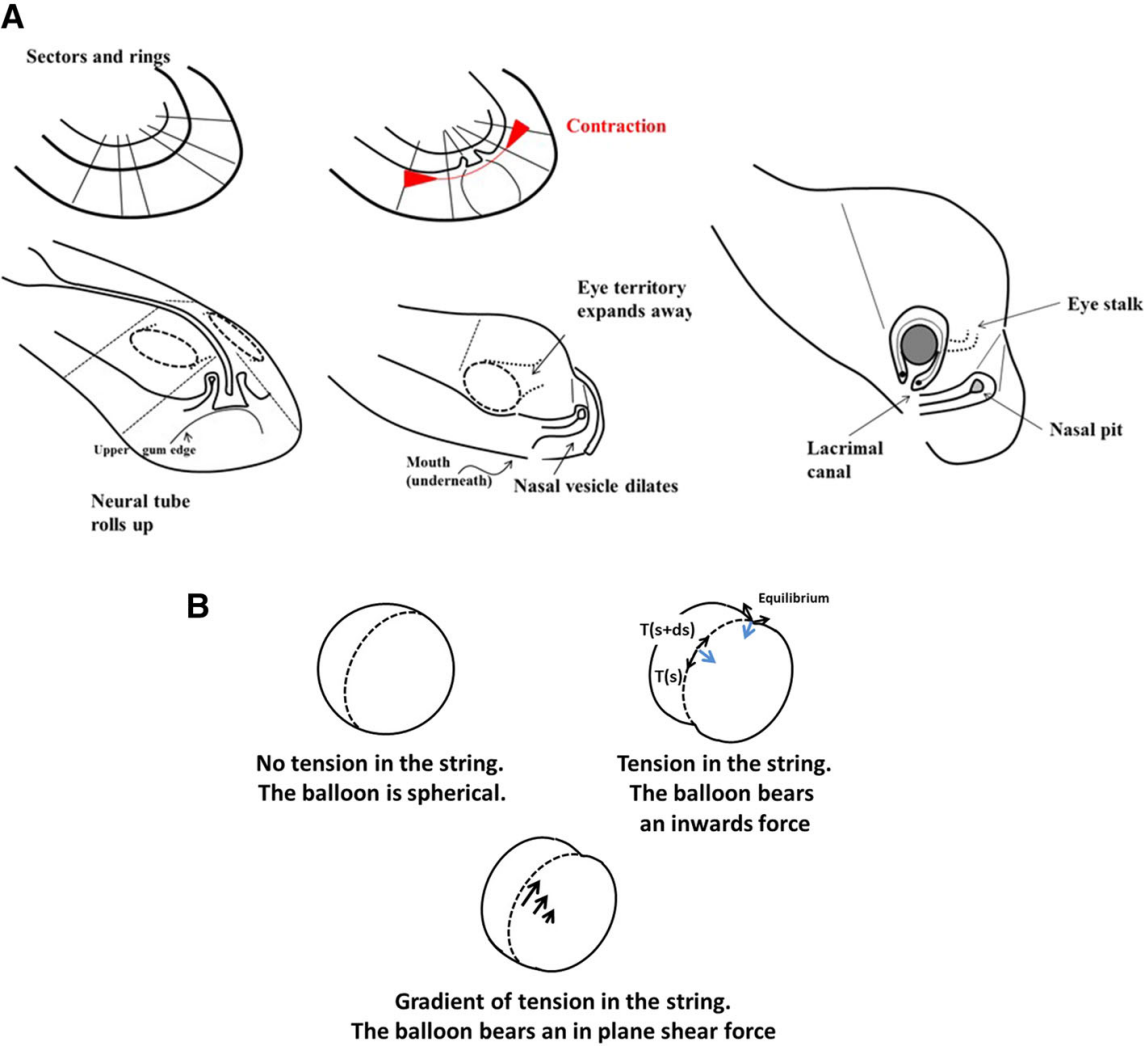

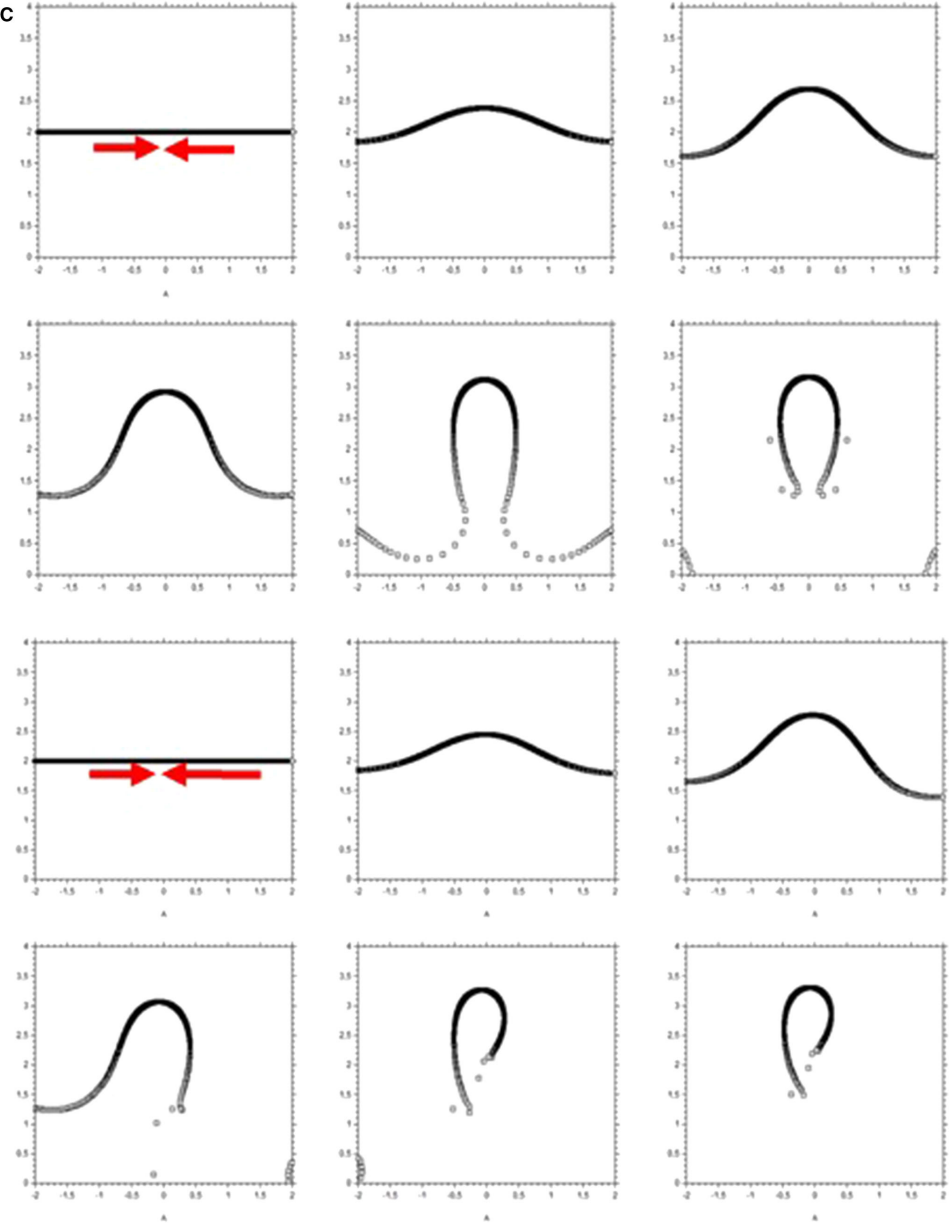
Mechanism of formation of the nose complex. **a** Initially, there is a structure in rings and sectors in the blastodisc (top left). The nasal territory and the eye territory are prepatterned by the lines. The D-V boundary contracts in the anterior area, the nasal sector flexes and forms a small hairpin on the surface along the presumptive mouth boundary. It forms an elongated tong of tissue converging towards the median line. During neurulation, the pattern rolls up (bottom left). The front edge flexes down and forms the mouth edge underneath (the gum actually). As the brain vesicles swell, two furrows form on the dilating brain corresponding to the cables locking the eye and nose territories. The eye invaginates: the corner of the eye finds itself directly connected to the basis of the nose territory (Bottom Right). The nose invaginates in a hairpin pattern, because it is a hairpin or horseshoe in the first place. **b** Explanation of the effect of tension in a string or cable acting against dilation of a vesicle. If there is no tension in the string, the surface tension of the balloon is isotropic and the balloon is spherical (top left). If there is tension along a string, the line tension induces an inward force related to the derivative of the tangent vector (top right, blue arrow). This inward force is equilibrated by the inverted curvature of the balloon in the furrow. However, if the tension itself varies, there is an additional tangent force which induces shear (bottom), this is akin to a Marangoni effect. **c** Top Modelling of horseshoe formation by a simple viscous potential flow model. We assume that the D-V boundary constricts (quadrupolar system of forces [3]). We assume that the locking by the cables orients the gradient of tension away from the cable (stated otherwise, the cable region is less deformable). If we take a D-V boundary and deform it visco-elastically in a symmetrical contraction force (red arrows of same length), we get a symmetrical winding which tends to form a horseshoe or hairpin (Video 28). **c** Bottom We now assume that the contraction is stronger frontally, since the nose territory is located sideways, where it neighbours the eye territory (see Fig. 2b), we expect the *bilan* of forces to be asymmetrical (asymmetrical red arrows). If we deform a boundary in such an asymmetrical pattern of forces (viscous flow), we form a skewed finger evoking the bent slits seen in the dogfish for example. During formation of the horseshoe, a concavity forms on the other side (star in** b** top) which is another niche for possible nare formation, explaining well the situation observed in the thornback ray (Fig. 1b, bottom). The mathematical model for ** c** is detailed in Ref. [3]

We have observed that during neurulation, closure of the notopore, likely by preventing fluid escape, causes or at least contributes to cause the strong dilation of the brain vesicles. The dilation of the vesicle, the continued contraction of the mouth area, and the pull along the nasal sector hardly deforms the nasal area which remains a horseshoe at the apex of a U-turn or hairpin of tissue. This is how the nasal pit territory forms; Fig. 12a summarizes schematically the process. These geometrical facts explain readily why the placode is not circular, and why it is a horseshoe inserted midway inside a sector. Also, if nose formation occurs earlier during the morphogenetic movements, the oculo-nasal area will be morphologically different: at early stages, it will look for example as the dogfish olfactory system. Early stages of chicken development are reminiscent of what is seen in fish. If the furrow of the hairpin does not heal, a nose-to-mouth slit will be apparent.

The tension in the strings or cables (the radial lines or rays seen inside the blastula) explains readily the presence of the valleys separating the brain vesicles (Fig. 12b). Indeed, a linear tension T**t** along a curved line with tangent vector **t** imparts an inward force T**n**/R by Laplace formula, in which **n** is the normal vector; this is the force which hinders the ballooning of the vesicles. This inward force is equilibrated by the inverted curvature in the direction perpendicular to the valley direction (Fig. 12b top right). Now, in addition, we also observed in Fig. 8 a linear force along the furrow generating a shear force. This linear force is what would be expected for a non-uniform tension, with a gradient. Indeed, a gradient of linear tension generates an in-plane force ∂s**T**(s) = ∂sT(s)**t** along the curvilinear direction (Fig. 12b bottom). It has been shown in Ref. 17 that the early embryonic movements proceed by a gradient of tension which generates dipolar and quadrupolar movements. Such a gradient of tension generates a force which orients visco-elastic movements towards the corner of the mouth. This is why, as the mouth territory constricts, both the nasal area and the eye area point and converge in that direction. Therefore, we find a common explanation to the localization of the nasal pit, its form, its orientation, its correlation with mouth and eye formation, and the presence of long-ranged valleys occupied by tissue cables and defining the brain vesicles. Of course, depending on the magnitude of the forces, the pattern of convergence of the nose the eyes and mouth, encapsulated in the shape of the nasogenian groove, will be more or less acute.

The spurious contraction twitches and the deeper contractions which follow the surface contractions show that the tissue is contractile and active, that the contractility is latent and excitable, and that the spatial pattern of the contraction is congruent with the geometry. This excitability or latent contractility provides also a mechanism explaining why some culture media are able to trigger rapid nasal pit formation [30], since contractions are sensitive to ionic concentrations, and ATP levels. In the same spirit, we have shown in Ref. 29 that, during limb development, embryonic tissue contraction has a pattern which follows tissue geometry. Here the hairpin of tissue contracts following the existing prepattern, in a similar fashion as peristaltic waves along the lung [31] or the gut [32]. The propagation of the contraction along the nasal hairpin triggers the formation of the nose by contraction of the edge of the hairpin.

One important aspect of the mechanism of ectoderm delamination and buckling lies in the fact that the excitability is not just in plane but also out-of-plane. As shown in Videos 24, 25, 26, the twitches propagate in depth and stimulate contraction of the underneath cell layers. In plane, the tissue is close to continuous, but it is discrete out of plane (there exist stratified cell layers). Since the tissue layers are triggered in a discrete sequence, the propagation from one layer to the next happens in sequence, with some discrete delay. This causes a strain mismatch between layers (one layer starts to constrict, while the other is already relaxing), thus favouring delamination and roll-up of the nare ridge (delamination inside the nare area is manifested by a double nare edge shortly visible in Video 20). Since, in addition, there is a sector of tissue stretched anisotropically in the oral to dorsal direction, delamination and invagination occurs sooner in the top part of the horseshoe forming the initial nasal pit cavity, as observed.

The observations reported here explain also in a straightforward manner why there exists a lacrimal canal between the eye and the nose. The canal connects the nares to the U-turn fold of the eye cupula (the fornix), because they correspond remotely to neighbouring areas in the corner of the mouth (Fig. 2b, see also Video 9), and they are both dragged towards each other by tissue contraction in the corner of the mouth area.

The taxonomy shows the interesting case of the lampreys, and other jawless fish. The agnatha have one single nostril, along the median axis (Fig. 1b Top), in between the eyes [8, 9, 20]. It appears that the single nostril of the lamprey is in fact the semi-hole formed by the U-turn or median fornix at the apex of the neural tube (anterior notopore), visible, for example, in our Video 22 in frontal view, or in Fig. 6a, top left. This shows that U-turn fornices such as the eye fornix or the notopore are favourable spots for organ development, being an almost closed hollow niche. In most vertebrates, this area locks the pituitary gland [20].

One may wonder why there is a bifurcation between a single nostril along the median axis, or two on either sides, and whether this is a mere evolutionary coincidence happening by hazard, or a true morphogenetic bifurcation. Systems biology would invoke some induction loop selected at random. Physics would rather search for a driving parameter causing some bifurcation. We have observed that closure of the notopore (nasal pit in the lamprey) enhances brain dilation. We also observed that there is a tension force in the furrows oriented rostrally. We see that if the rostral force in the threads or cables is increased, the head rocks forward more (as in Video 27). Increasing the linear tension along the median axis pulls on the notopore; then the notopore is stretched in a direction which increases the likelihood of notopore closure (the median force being increased, the neural folds tend to close more tightly, as when pulling on a rubber foil, see Fig. 3b in Ref. 4), but conversely, the increased pull in the cables is associated to an increased pull forming a more pronounced hairpin which favours the formation of paired nostrils. This comes from the fact that, while out-of-plane buckling is enhanced by traction, in plane deformation is enhanced too. Hence, there is a dynamic rationale to the transition from agnatha (no jaw, one single nostril), to gnathostomes (a jaw and paired nostrils), by increase of the linear force in the cables (a one dimensional parameter acting simultaneously on head flexure and nostril prepatterning).

Moreover, if we return to the picture of the thornback ray (Fig. 1b Bottom), we see that the four nares are actually associated to the convex side and the concave side of the nasal sector, deformed in a hairpin manner, each side of the fold corresponding to an almost closed niche.

While all these movements may seem awfully complex and beyond any mathematical analysis, a parsimonious physical picture can nevertheless be given (Fig. 12c, Video 28). The presumptive nasal territory finds itself at the oral boundary (represented by the black line). The contraction of the DV boundary advects visco-elastically the entire tissue. In a very crude approximation, we assume a contraction force along the oral boundary [3, 4], modelled by a force term aligned at the D-V boundary (red arrows). By flow conservation, it is equivalent to assume a contraction along the D-V boundary or to assume a traction at right angle, along the nasal sector (this, by the way, constrains the developmental process). If we assume a symmetrical contraction, we find generic deformations as in Fig. 12c (top). But since the nasal territory is not along the median axis, we may introduce an asymmetry of the quadrupolar flow with a gradient of contraction oriented towards the median axis, specified by two dipolar forces one of higher magnitude along the median axis. Thus, we find typical generic deformations as in Fig. 12c (bottom). In all cases, we find horseshoe shapes oriented towards the oral line, with a secondary lateral concavity corresponding to the second nare in fish with four nares.

## Conclusion

This works shows that face dynamics in the chordate phylum is not at all arbitrary. It is prepatterned by a set of contracting lines forming rings and sectors. These rings and sectors are related to the early pattern of zygote cleavage [3, 4]. The dynamics of morphogenesis is actually latent in the geometry of cell division, which is biaxial (the sequence of cell cleavages occurs at right angles). There is a strong coupling between geometry and dynamics, both in-plane and out-of-plane. Cells tend to align in the existing pattern, under in-plane traction forces and form cables. The lines of the pattern behave as tiny pulling strings. In the first place, cell cleavage occurs by contractions, which prepattern this set of strings.

We have shown that in the early embryo, the contraction of the tissue is both in-plane and out-of-plane. The out-of-plane component of the contractions creates out-of-phase strains which favour delamination in a stratified biological tissue. All this renders animal body formation, with its sensory organs, a robust dynamical process explaining the generic features of vertebrates face, and the fact that sensory organs are connected to the brain. In the specific case of the nose, the pattern cannot be reduced to a round placode; there is a holistic aspect to nose formation, with simultaneously a canal towards the eye, a slit towards the mouth, and a likely physical bifurcation related to force actuation, all related to the dynamics of contraction in a pattern of radial and orthoradial lines. Along these lines, contraction forces are long ranged and they induce holistic features such as grooves or furrows which are readily observable on any face. The molecular cause of these contractions is likely to be the actin-myosin complex, which causes contraction of rings at early embryonic stages [34, 35]. More generally, the lines present in the early blastula serve as mechanical cues for the entire morphogenetic process, and they connect the sensory organs to the inside of the brain. The dynamics explained here may help to clarify certain teratologies such as holoprosencephaly [6]. These face teratologies show a continuous spectrum of abnormalities, ranging from almost normal face with a mild narrowing of the inter-ocular distance, to a very narrow front with cyclopia, and absence of nose. It makes sense that since the initial morphogenetic movements comprise a contraction of the oculo-naso-oral area along a circular line running around the dorso-ventral boundary and connecting the mouth, the nares, the eyes and even the ears, a smaller or larger traction force along such a line will lead *in fine* to faces with eyes and nares more or less asunder.

## Supplementary Information

Below is the link to the electronic supplementary material.

Supplementary file1 (DOCX 527 KB)

Supplementary file2 (AVI 19936 KB)

Supplementary file3 (AVI 32073 KB)

Supplementary file4 (AVI 24615 KB)

Supplementary file5 (AVI 18065 KB)

Supplementary file6 (AVI 15949 KB)

Supplementary file7 (AVI 6968 KB)

Supplementary file8 (AVI 26011 KB)

Supplementary file9 (AVI 6797 KB)

Supplementary file10 (AVI 13759 KB)

Supplementary file11 (AVI 7831 KB)

Supplementary file12 (AVI 49506 KB)

Supplementary file13 (AVI 6229 KB)

Supplementary file14 (AVI 8805 KB)

Supplementary file15 (AVI 8213 KB)

Supplementary file16 (AVI 24849 KB)

Supplementary file17 (AVI 7388 KB)

Supplementary file18 (AVI 12381 KB)

Supplementary file19 (AVI 11169 KB)

Supplementary file20 (AVI 13369 KB)

Supplementary file21 (AVI 12158 KB)

Supplementary file22 (AVI 9854 KB)

Supplementary file23 (AVI 8315 KB)

Supplementary file24 (AVI 9307 KB)

Supplementary file25 (AVI 19265 KB)

Supplementary file26 (AVI 8805 KB)

Supplementary file27 (AVI 4439 KB)

Supplementary file28 (AVI 1981 KB)

Supplementary file29 (AVI 17319 KB)

## Data Availability

All the data, mostly high-definition time-lapse videos of development, are freely available upon request by the author. The custom Java plugins for tissue tracking, and strain tensor analysis are freely available upon request.

## Footnotes

1 The main groove on the face on either side of the nose.
