# Supplementary material for: Dynamics of early stages of nose morphogenesis
Source: Eur Phys J E Soft Matter. 2022 Nov 19;45(11):93. doi: 10.1140/epje/s10189-022-00245-8 (PMC9674774; doi:10.1140/epje/s10189-022-00245-8)
Supplement: Supplementary file 1 — Supplementary file1 (DOCX 527 KB) [file 10189_2022_245_MOESM1_ESM.docx]

**Supplementary Material Methods.**

We describe first the preparation of the embryos (Supp. Fig. 2). For study of nasal pit formation, the eggs were most generally incubated in the evening by 6pm so that they were ready for the experiments in the morning of the 3^rd^ day, counting the first day of incubation as zero (for example if eggs are put to incubate on a Friday evening, they are ready on Monday morning, contraction will start by noon, and the nasal pit will form in the afternoon). The shell is broken and the content is dropped into a square plastic cup. If the egg is handled carefully, always keeping the same orientation, the embryo lays generally on top, because of buoyancy of the yolk over the albumin. The embryo is removed from the egg by cutting a round patch of the vitelline membrane around the yolk-sac and embryo (Supp. Fig. 2). These are transferred to a dish with a spoon. There, all the yolk and albumin is rinsed off with Phosphate Buffer Saline (PBS from Dulbecco), and a pipette, and the vitelline membrane is removed with fine tweezers. By day 2.5, the embryo starts to be wrapped by the amniotic sac. In order to improve imaging, the amniotic sac must be removed. In this case, the amniotic sac is torn apart with fine tweezers. The left side of the amniotic sac is more difficult to dissect off being almost stuck to the embryo head. Next the embryo and its yolk-sac are laid flat in a Petri dish, in approximately one centimeter of PBS with the dorsal side down. Next, the anterior part of the yolk-sac is pulled posteriorly with tweezers. By so doing, the head appears now facing the observer, over a dark background. At the end of these steps, the underneath part of the head is more visible, except at stages close to nasal pit formation, when head flexure renders nasal area still more difficult to see. At that stage, we cut a thin plastic thread from a plastic cup (Supp. Fig. 2 Bottom), and slide it gently under the neck, starting from the dorsal side of the neck. By so doing, the head is now lift off slightly, such that the presumptive nasal area appears more clearly. One may think that after all these steps the embryo should be dead. This not the case, and even in the absence of culture medium, the embryo will develop for 6 to 15 hours. It is remarkable how robust the embryos are. The embryo is imaged in white light with a Schott 1500W fiber lamp, either under a binocular (Macrofluo or FLZ III from Leica), or an upright microscope (Nikon Eclipse). A two slits metal plate is used which enhances the contrast by a Mac-Zender type interferometric effect (the two slits system provides a dual light beam, by reflection on the edges, which interfere and increase contrast). The camera was either a monochrome Stingray 200 interfaced with AVT Smartview, or a Basler CMOS camera interfaced with Phase gmbh plugin in ImageJ (ImageJ software from Wayne Rasband), one movie was acquired with an older analog camera from Watek, interfaced with a Scion Frame Grabber DAC board (discontinued). This has allowed me to film the formation of the nasal area starting from the blastula stage. For the sake of completion, and for the final discussion, I give also Videos 12 and 20 which show eye and ear formations as obtained with this technique. The video 20 was presented elsewhere in the context of ear studies [3].

**Supplementary Material Figures**


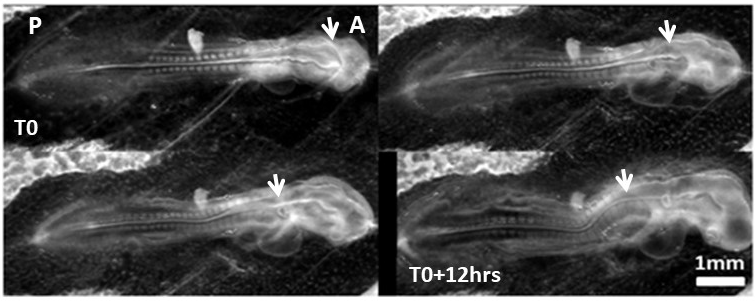


**Supplementary Material Figure S1**

**Supplementary Material Figure S1 Head flexure impedes observation of the nasal territory.**

Around the moment of sensory organs formation (HH stages 13-18), the chicken head undergoes a pronounced forward flexure which renders nasal area imaging quite difficult. In addition, the embryo is wrapped by the chorio-amniotic fold (arrows) which blurs the image. This is why the embryo must first of all be removed from the amniotic sac, and next oriented in a different position, in order to get a meaningful image. (A : Anterior ; P : Posterior).


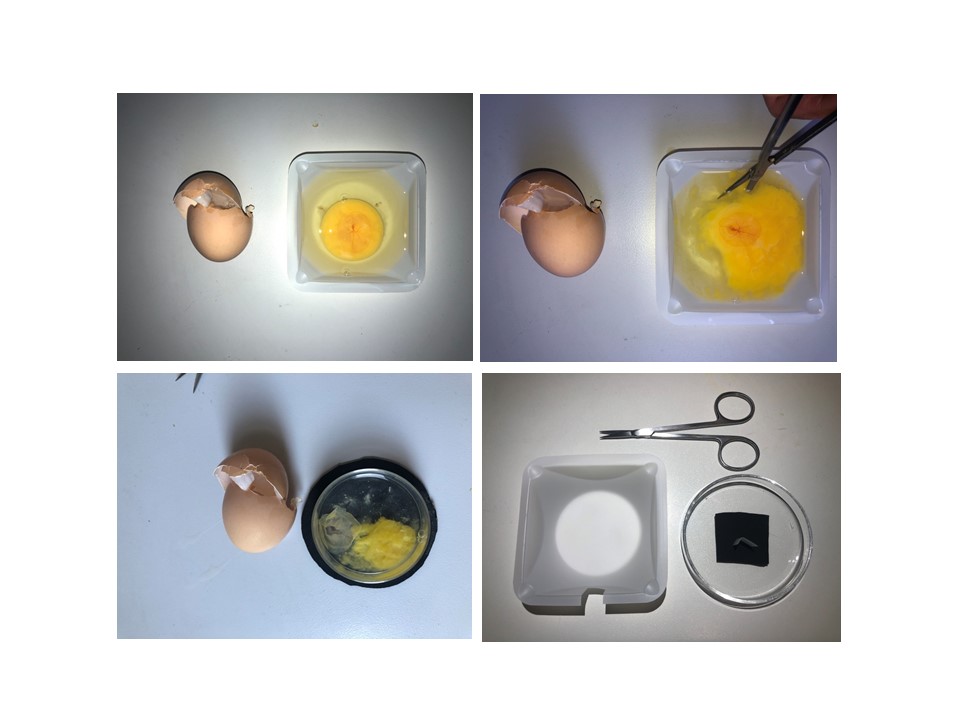
**
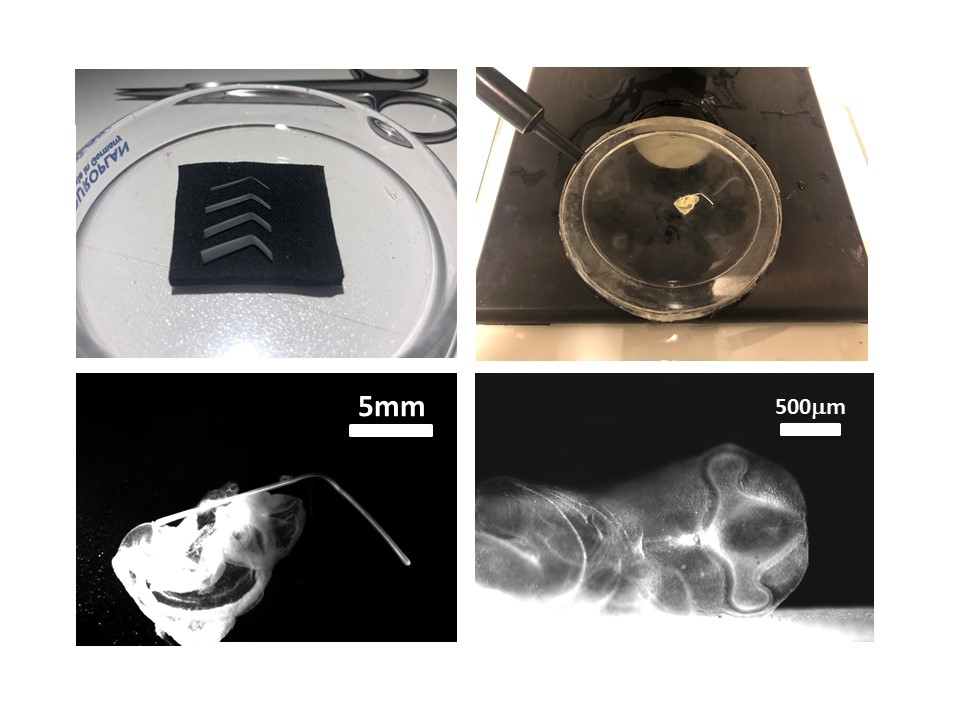
**

**Supplementary Material Figure S2**

**Supplementary Material Figure S2 Steps in preparing embryos for nasal territory observation.** The egg is cracked in a plastic cup. The embryo is cut off and transferred to a Petri dish ; there, it is rinsed. A plastic thread is cut from the angular part of a similar plastic cup. Several threads can be cut, of different widths, to tilt more or less the embryo head. After rinsing, the embryo is turned, the body is lifted and positioned along the edge, so that the head hangs away. This allows one to image more properly the nasal area. Here, the embryo is oriented in a completely frontal view (the embryo is alive, its heart beats properly).


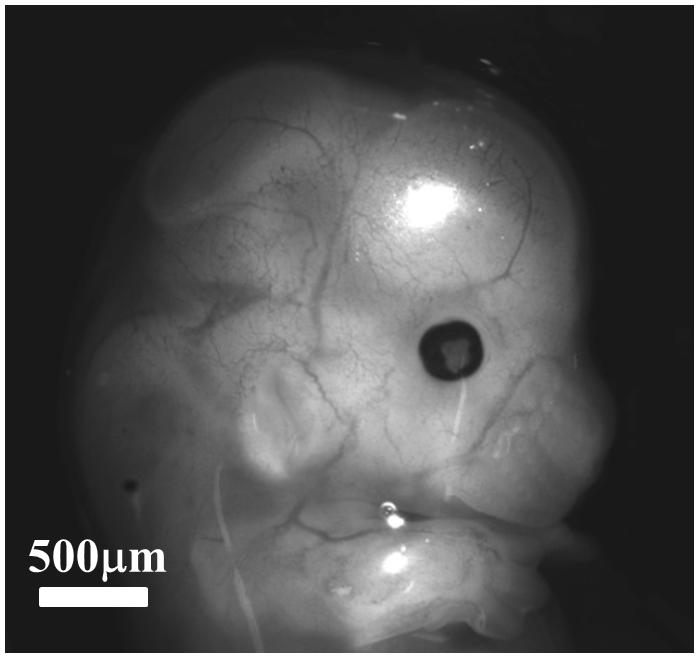


**Supplementary Figure S3** During embryo development it is a classical observation that the main blood vessels are positioned in the valleys of the brain vesicles (here a mouse embryo E13.5). Especially, to the right of the eye, one sees the blood vessel which follows the valley separating the ocular territory and the nasal territory.

**Supplementary Material Particle Imaging Velocimetry (PIV)**

Displacements were extracted from the Time-Lapse videos by Particle Imaging Velocimetry (PIV). PIV is a now classical numerical tool to extract movements [35]. It works by correlation function between selected domains which are tracked from one frame in a stack to the next. Concretely, we have a macro in ImageJ developping language (“Selectagrid”) which allows one to generate at will a grid of points with desired spacing. We next apply the plugin “Tracker” (courtesy of O. Cardoso and B. Abou). The “Tracker” pluggin generates by correlation function the file containing the positions (M_x_, M_y_) of the points as followed in each frame (Lagrangian view of the flow, the data set is the trajectory). From this file we reconstruct the 2D-strain field by calculating the displacements (u_x_,u_y_) and the components of the strain tensor ε_xx_=∂_x_u_x_, ε_xy_=1/2(∂_y_u_x_+∂_x_u_Y_), ε_yy_=∂_y_u_y_. The 2x2 strain tensor is diagonalized point by point with standard algebra. The eigen vectors correspond to the directions of principal strain, the eigenvalues correspond to principal extensions. A cross is drawn at each point oriented along the principal axis of strain, and the arms of the cross have lengths proportional to the eigen values (length ¾ of the interspacing for an eigenvalue of 1). This plugin (author V. Fleury) is freely available upon request.

**Supplementary Material Videos**

**Video 1** Time lapse video-microscopy of a 2 days embryo observed shell less at low magnification under a binocular. One sees the flexure of the head, the formation of the ear pit and eye, and the chorio-amniotic fold which descends along the body to finally wrap the embryo. Magnification 0.7X, dorsal view, duration 12hours.

**Video 2** Time lapse video-microscopy of a 1.5 days embryo observed shell less at magnification 4X. Physiologically the embryo develops the ventral side down. One observes the pull by the primitive streak which triggers the folding of the blastula and chord extension (the morphogenetic force is located dorsally). In the anterior part the eye vesicles appear as the neural folds get into contact. The nasal area is impossible to image in this configuration, since it is located in the anterior part, somewhat ventrally (see Supplementary Fig. 1). The first somites (vertebrate precursors) are visible to the right. If one observes carefully the neural tube, one may see the lines corresponding to the ocular sector which are stretched in the posterior direction. Dorsal view, duration 13hrs.

**Video 3** Time lapse video-microscopy of early stage of embryogenesis showing the contraction of the anterior sector of the blastula, prior to neurulation (Mag. 10X, data for Fig. 2). The rings corresponding to the neural territory the dorsal territory, the ventral territory and the extra embryonic organs are clearly visible (white light, no staining). The phenomenon was particularly visible in this embryo. As the mouth sector located to the left in this movie starts to deform in the contraction movement, one may see the two folds which are going, eventually, to form the nares (arrows). (Dorsal view, duration 7 hrs).

**Video 4** Time lapse of blastula contraction showing the earliest appearance of the ocular territory at the onset of neurulation (Mag. 4X, data for Fig. 3). The anterior sector will be the mouth sector. The next visible sector is the nasal sector and next the ocular sector. The edge of the mouth sector coincides with the limit of the nasal and ocular territories. To the right a magnified view of the presumptive ocular area showing the contraction of a horseshoe sector corresponding to the eye territory. (Dorsal view, duration 9 hrs).

**Video 5** Beginning of neurulation (Mag. 10X, data for Fig. 4A). Time-lapse showing the edge of the eye sector coinciding with the kink in the neural fold marking the anterior limit of the eye territory (dorsal view, duration 8hrs).

**Video 6** Time-lapse video microscopy (Mag. 10X, data for Fig. 4B) of embryo neurulation and early stage of eye stalk extension. By the end of the video the neural crest cells start to migrate. In the first frames one sees the anterior valley or kink in the neural tube corresponding to the limit of the eye territory. (Dorsal view, duration 8hrs).

**Video 7** Time-lapse video microscopy (Mag. 10X, data for Fig. 5). Onset of lateral extension of the eye stalk showing the flattening of the presumptive ocular tissue and its lateral winding (dorsal view, duration 2hrs).

**Video 8** Time-Lapse video microscopy at Mag. 4X showing the lateral expansion of the eye stalk. (Dorsal view, duration 12hrs).

**Video 9**  Time-lapse video-microscopy at Mag. 4X showing the lateral expansion of the eye capsule and brain dilation. The nasal territory is found below the eye capsule; this stage is approximately the same as the one in dorsal view in Video 8. (Ventral view, duration 8 hrs).

**Video 10**  Time-lapse video-microscopy at Mag. 5X and 10X (to the right) showing the thin nasal hairpin between the ocular and oral territories. (Ventral view, duration 8hrs).

**Video 11** Time- lapse video microscopy at Mag. 4X showing the lateral expansion of the eye stalk and formation of the eye placode by flattening of the eye stalk against the superficial ectoderm. (dorsal view, duration 4.5hrs).

**Video 12** Time-lapse video microscopy at Mag. 10X, around the exact moment of notopore closure showing an acceleration of eye stalk extension and brain vesicle dilation (see Fig. 5B). (Semi-ventro-lateral view, duration 2hrs).

**Video 13** Time-lapse video microscopy at magnification 4X showing the ballooning of the brain vesicle as seen from a ventral view (data for Fig. 8). Please note the beginning of the ballooning of the nasal vesicle. (Ventral view, duration 7hrs).

**Video 14** Time-lapse video microscopy at magnification 5X of placode invagination forming the eye ball and the lens. One sees that the eye folds have an almond shape oriented frontally towards the nasal and oral area. (Frontal view, duration6hrs).

**Video 15** Time-lapse videomicroscopy at magnification 2X of nasal pit formation, as observed “from underneath” with a binocular. One sees movements associated to nasal pit appearance. The pit forms brutally with a nonlinearity associated to apparent stress relaxation (ventral view, duration 8hrs).

**Video 16** Time-lapse video microscopy at magnification 3X of nasal pit formation with a binocular (Leica). One sees movements associated to nasal pit appearance. A correlation between the nasal contraction and pit formation is evidenced.

**Video 17** Time-lapse video microscopy at magnification 4X of nasal pit formation with a microscope (Nikon Eclipse data for Fig. 7B). One sees movements associated to nasal pit appearance. A correlation between the nasal contraction and pit formation is evidenced, and a sector is evidenced above and below the nare (Profile view, duration 4 hrs).

**Video 18** Time lapse video-microscopy at magnification 10X of nasal pit formation with a microscope in white light (Nikon Eclipse, data for Fig. 7C). One sees movements associated to nasal pit appearance. A correlation between nasal contraction and pit formation is evidenced. An elongated territory is observed below the nare with a faintly visible furrow (see fig. 7C). The contracting nasal territory neighbours the eye fold. The territory below the nare will eventually form the lacrimal canal existing between the nose and the eye. This situation originates in the hairpin fold visible in Videos 3 and 10, separating the ocular and nasal territories. (Duration 7hrs).

**Video 19** Time-lapse video at magnification 10X of nasal pit formation. One sees a strong contraction of the nasal territory. In this Video, the opening of the nare and invagination of the nare ridge is visible by the end. The nasal sector is visible to the right of the nare (anterior is to the right). (Profile view, duration 5hrs)

**Video 20** Close up view of the nare movement after registration of the frames in Video 19. One observes a strong contraction of the nasal territory, which correlates with a radial contraction of the very edge of the nare (narrowing of the ridge), which causes an opening of the nare (widening of the diameter). The superficial ectoderm is seen to invaginate at the ridge edge. (Mag. 10X, duration 5hrs).

**Video 21** Time-lapse video microscopy at magnification 4X-10X-4X of ear pit formation, with the same imaging technique. (Dorsal view, duration 10hrs).

**Video 22** Time-lapse video microscopy at magnification 4X of neural folds roll-up, in the area of the notopore (concatenates two embryos). The apex of the neural tube forms a U-turn fold which is discussed in the conclusion in reference to the lamprey nasal area. By the end one sees on either side of the eye folds the nasal territory which forms a kink or wedge of tissue (which appears darker because it is thinner), lateral to the closure of the notopore along the median axis (arrow). (Frontal view, duration 10 hours).

**Video 23** Endogenous contraction twitch observed in the nasal area prior to nasal pit formation (other examples available, see Fig. 11A). (Semi-frontal/lateral view, duration 30Min.)

**Video 24** Endogenous contraction twitch observed in profile view, in the nasal area, just prior to nasal pit formation. The movement shows that the contraction twitch flexes the surface and stimulates the underneath layers (after the flexure, a movement starts underneath). Please magnify the video to full screen to see properly the contraction twitch. (Profile view, duration 30Min.)

**Video 25** Endogenous contraction twitch observed in profile view in the ocular area just prior to lens formation. The movement shows that the contraction twitch flexes the surface and stimulates the underneath layers (after the flexure, movement starts in the underneath layer). (Duration 30Min.)

**Video 26** Longer Time-lapse showing the onset of invagination, after the contraction twitch (Mag. 4X, Duration 2hrs).

**Video 27** Time-Lapse of head flexure at Mag. 4X. Head flexure correlates with mouth invagination (Duration 4 hrs).

**Video 28.** Animation of a line forming a nasal loop in a viscous flow. A line is advected in a contraction flow formed of two force dipoles oriented “head on”.
